# Lumbar Disc Herniation with Contralateral Symptoms: A Case‐Series of 11 Patients and Literature Review

**DOI:** 10.1111/os.13849

**Published:** 2023-09-25

**Authors:** Qingyang Gao, Huiliang Yang, Umar Masood, Chunguang Zhou, Ying Cen, Yueming Song

**Affiliations:** ^1^ Department of Plastic and Burn Surgery, West China Hospital Sichuan University Chengdu China; ^2^ Department of Orthopedics, Orthopedic Research Institute, West China Hospital Sichuan University Chengdu China; ^3^ Jacobs School of Medicine and Biomedical Sciences University at Buffalo, The State University of New York Buffalo New York USA

**Keywords:** Diskectomy, Intervertebral Disc Degeneration, Laminectomy, Sciatica

## Abstract

**Objective:**

Lumbar disc herniation (LDH) is a common pathology that typically causes unilateral radiculopathy on the same side as herniation, while patients may occasionally present with contralateral symptoms. Owing to the rare incidence of LDH with contralateral symptoms, the pathological mechanism remains unclear and the optimal surgical strategy is a subject of debate. This study aimed to provide new insights into the pathological mechanism of contralateral symptoms and assess the efficacy of ipsilateral hemilaminectomy and discectomy surgery in this population.

**Methods:**

This study was a retrospective, single‐center, clinical case series, including 11 LDH cases with exclusive contralateral symptoms. We searched for LDH cases that were presented at our institution between January 2011 and December 2020. Adult LDH Patients with contralateral radicular pains were included, while those with ipsilateral radiculopathy, lumbar stenosis, foraminal stenosis on the symptomatic side, multilevel disc herniations, scoliosis, and lumbar operation history were excluded. Visual Analog Scale (VAS), clinical features, radiographic images, and other data were collected from the study cohort of 11 cases for further analysis. We also reviewed LDH cases in English literature from 1978 to 2023 to analyze their clinical characteristics and treatment.

**Results:**

The incidence rate of LDH with contralateral symptoms in single‐level LDH cases was 0.32%. The average age of our 11 cases was 49.3 years old, and five of them were female (45.5%). All individuals had single‐level lateral LDH, with six cases (54.5%) located at L4‐5 and five cases (45.5%) located at L5‐S1. Upon admission, patients presented with lower back pain (seven cases, 63.6%), radicular pain (seven cases, 63.6%), hypoesthesia (seven cases, 63.6%), and muscle weakness (one case, 9.1%) on the contralateral side alone. Each case experienced ipsilateral hemilaminectomy and discectomy, and no lateral recess stenosis, hypertrophy of facets or ligaments, and sequestrated discs were found during surgery. All of them have good pain relief with two cases reporting no pain and nine cases reporting only mild pain at the last follow‐up.

**Conclusions:**

Based on the surgical findings of our 11 LDH cases with contralateral symptoms, we hypothesized that the contralateral symptoms might be produced when the nerve root on the contralateral symptomatic side was tightly pulled by the herniated disc via the dural mater. Ipsilateral hemilaminectomy and discectomy surgery effectively and efficiently relieve the symptoms without postoperative complications for these patients.

## Graphical abstract

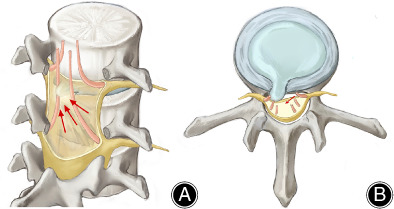

## Introduction

Lumbar disc herniation (LDH) is a common degenerative musculoskeletal pathology. It can be associated with aging, trauma, genetics, and congenital abnormalities.^1^ Radiculopathy is a dominant feature of LDH. The lumbar spinal nerve roots are compressed by the adjacent herniated intervertebral disc, leading to radicular pain or numbness in the unilateral limb ipsilateral to the disc herniation.^2^ Moreover, patients with LDH can also present with back pain^3^ and cauda equina syndrome (CES).^4^ Most patients with radical pain only respond to conservative treatment, while some patients may undergo laminectomy and discectomy to relieve the pain.^5^

Consistent with the anatomical relationship between intervertebral discs and spinal nerve roots, LDH with contralateral symptoms is very rare, with only a few cases reported in the literature.^6^, ^7^, ^8^ Some cases develop contralateral radiculopathy on the non‐lesion side, without any symptoms or abnormal signs on the disc herniation side. There seems to be unidentified pathological mechanisms of the contralateral symptoms. Some hypotheses have been proposed to explain the occurrence of contralateral symptoms, including nerve root traction,^7^ epidural fat,^9^ and venous congestion.^6^ But, these factors posited in the literature do not fully explain the lack of ipsilateral symptoms.

Due to the inconformity between symptoms and radiographic findings, the surgical approach remains controversial. Three surgical strategies have been proposed in the literature: (1) unilateral approach and discectomy on the side of the patient's symptoms^10^, ^11^; (2) unilateral approach and discectomy on the side of the disc herniation^7^, ^12^, ^13^, ^14^, ^15^, ^16^, ^17^; and (3) bilateral laminectomy and discectomy.^10^, ^15^, ^18^, ^19^, ^20^, ^21^, ^22^, ^23^, ^24^ Compared with the unilateral approach, a bilateral laminectomy will increase the operation time and complication risks. However, it is challenging to ensure the relief of symptoms via a unilateral approach through performing decompression on the symptomatic or disc‐herniation side.

The purpose of our study is to discuss the prevalence of this condition, investigate the pathological mechanism by which the symptomatic side and disc‐herniation side are mismatched, and subsequently, determine whether unilateral approach laminectomy and discectomy on the disc‐herniation side could successfully eliminate contralateral radicular symptoms.

## Methods

### Study Design and Subjects

**FIGURE 1:**
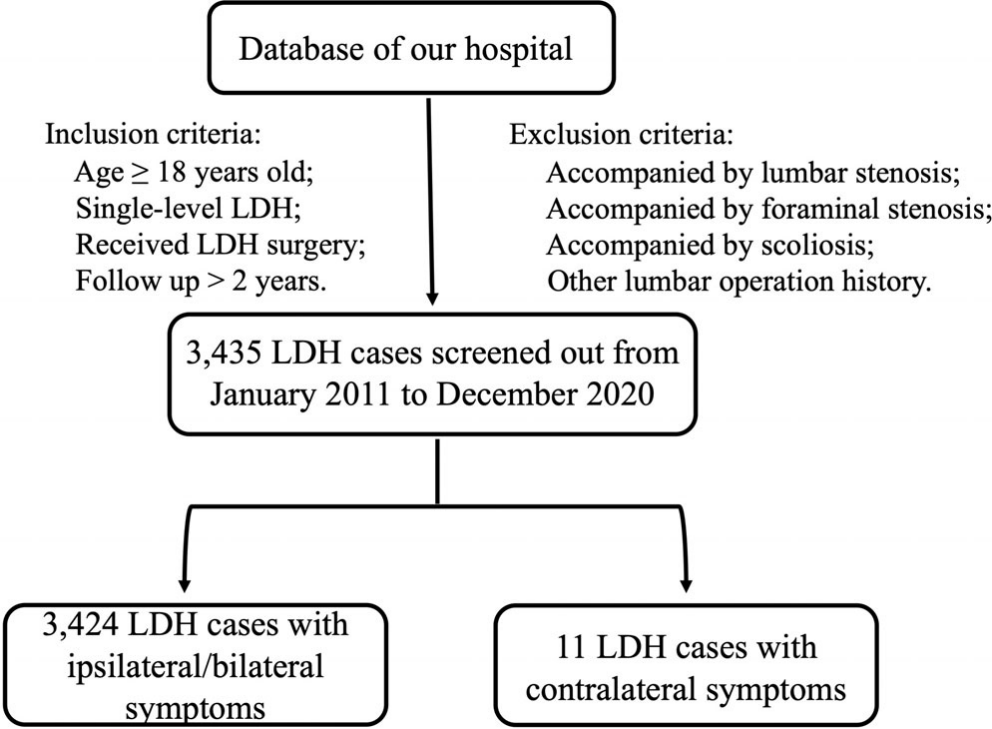
Flowchart of patients' selection process. (LDH, lumbar disc herniation).

This retrospective study was approved by our institutional review board. In the preliminary screening, we searched for all LDH patients admitted to our hospital system between January 2011 and December 2020. Next, we included 3435 patients with the criteria as followed: (1) single‐level LDH cases were confirmed by the corresponding segment computed tomography (CT) or magnetic resonance imaging (MRI) patients were defined by radiographic test; (2) ≧18 years old; (3) complaint of symptoms in the lower limb contralateral to the intervertebral disc hernia; (4) patients undergoing hemilaminectomy and discectomy surgery; (5) follow up ≧2 years. Cases of lumbar stenosis, foraminal stenosis on the symptomatic side, multilevel disc herniations, scoliosis, and lumbar operation history were excluded from the study (Figure 1).

LDH patients who presented with CES or severe neurological deficits were recommended emergency surgery. In addition, selective surgery was considered in cases where the medical history exceeded 3 months, conservative treatments proved ineffective, or if the patient experienced unbearable symptoms with evident disc herniation. Conservative treatments in our cohort included bed rest, anti‐inflammation medication, and physiotherapy. Postoperative patients received standard pain management and were encouraged to exercise early with a brace.

### Outcome and Other Variables

We reviewed patient electronic medical records for the 11 cases, from which the clinical, surgical, and treatment features were extracted. The following data were collected from the patient case files: age, sex, herniation side, symptom side, herniation level, symptoms and signs, symptom duration, preoperative Visual Analog Scale (VAS), surgery type, surgical findings, and postoperative complications. Postoperative VAS, follow‐up time, and long‐term complications were obtained from outpatient follow‐up or telephone follow‐up.

Lower limb radicular symptoms typically present as pain, weakness, or numbness along the affected nerve, which starts from the buttock and then radiates descending to the thigh, leg, or foot. A positive (<60°) Straight Leg Raising Test (SLR) is also an indicator of radicular irritation. Referring to the VAS score, the pain was graded as mild (VAS 0–2), moderate (VAS 3–6), or severe (VAS 7–10).

### Literature Review

**FIGURE 2:**
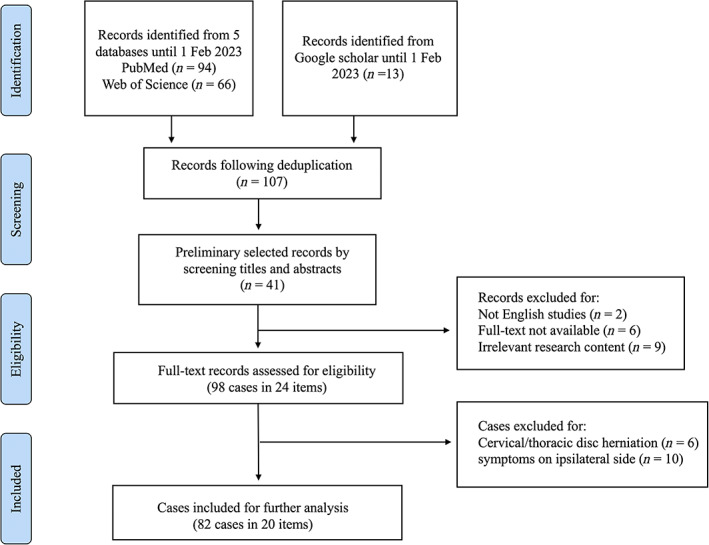
Flowchart of the study selection process.

We conducted a literature search in PubMed, Web of Science, and Google Academic from the date of establishment of each database to February 1, 2023 (Figure 2).

The search strategy for PubMed was: (((lumbar[Title/Abstract]) OR (lumbosacral[Title/Abstract])) AND ((disc herniation[Title/Abstract]) OR (herniated disc[Title/Abstract]) OR (Herniated lumbar disc[Title/Abstract]))) AND ((((((((((radiculopathy[Title/Abstract]) OR (radicular symptoms[Title/Abstract])) OR (leg pain[Title/Abstract])) OR (sciatica[Title/Abstract])) OR (sciatic pain[Title/Abstract])) OR (symptom[Title/Abstract])) OR (pain[Title/Abstract])) OR (lower limb pain[Title/Abstract])) AND ((contralateral[Title/Abstract]) OR (opposite[Title/Abstract]))).

We also searched the Web of Science and Google Scholar for additional sources with the keyword “contralateral symptoms” and “disc herniation.” The initial search identified a total of 107 publications. Ninety‐four articles were retrieved from PubMed, 66 articles were retrieved from Web of Science, and 20 articles were retrieved from the search engine. After the manual screening of titles and abstracts, 66 items were removed as irrelevant content. Seventeen articles were screened out for full‐read text for non‐English (*n* = 3), non‐full text (*n* = 6), or irrelevant content (*n* = 9). We also ruled out the bilateral cases (*n* = 10). Eventually, 82 cases in 20 pieces of literature were obtained for in‐depth analysis (Figure 2 and Table S1).

### Statistic Analysis

Word and Excel 365 (Microsoft, Redmond, WA, USA) were utilized for collecting data and statistical analysis. Continuous variables were reported as mean (range). Categorical variables were expressed as absolute frequency (*n*) and relative frequency (%).

## Results

### Demographic Details

Eleven LDH patients with contralateral radicular symptoms were identified from 3435 single‐level LDH cases in this study, of which six were males and five were female (Figure 1). The incidence of LDH with contralateral radicular symptoms among single‐level LDH cases was 0.32%. The average age of the cases was 49.3 years (range 34–69). The average follow‐up time was 33.82 months, and the longest follow‐up time was 46 months.

### Symptoms and Signs

Upon admission, the patients reported radicular pain (7/11), numbness (7/11), and weakness (1/11) in the leg opposite to the disc herniation with a disease duration of more than 1 year (Table 2). The mean VAS score at baseline was 6.91 (range 5–8), including zero mild cases (0 to 3 VAS), four moderate cases (4 to 6 VAS), and seven severe cases (7 to 10 VAS) (Table 1). Seven patients were associated with additional lower back pain (Table 2) and no cases were presented with CES.

**TABLE 1:** Characteristics of our 11 lumbar disc herniation cases with contralateral symptoms.

Age/Sex	Level	Herniation Side	Symptom Side	Symptoms/Signs	Surgery	Follow Up (m)	Pre‐op VAS	Post‐op VAS
45/F	L4‐L5	Right	Left	LBP and left leg numbness for 2 years; SLRT (+)	Ipsilateral hemi‐laminectomy and discectomy	26	8	1
34/M	L5‐S1	Left	Right	LBP and right leg radicular pain for 4 years; SLRT (+)		27	7	1
56/M	L4‐L5	Left	Right	Right leg radicular pain and numbness for 3 years; SLRT (+)		46	8	3
35/M	L4‐L5	Right	Left	Left leg pain and numbness for 5 years; SLRT (+)		31	6	1
42/F	L5‐S1	Left	Right	LBP and right hip pain for 1 year; SLRT (+)		35	7	2
39/F	L4‐L5	Right	Left	LBP and left leg numbness for 1 year; SLRT (+)		31	5	0
50/M	L5‐S1	Left	Right	LBP and right leg radicular pain for 3 years; SLRT (+)		35	7	3
67/F	L4‐L5	Left	Right	Right leg radicular pain and numbness for 1 year; SLRT (+)		27	8	2
69/M	L4‐L5	Right	Left	Left leg radicular pain and numbness for 6 years; SLRT (+)		45	6	0
58/F	L5‐S1	Left	Right	LBP and right leg radicular pain for 5 years; SLRT (+)		32	8	2
47/M	L5‐S1	Left	Right	LBP and right leg numbness and weakness for 1 year; SLRT (+)		38	6	2

Abbreviations: F, female; L, lumbar vertebra; LBP, lower back pain; M, male; Post‐op, post‐operation; Pre‐op, pre‐operation; SLRT, straight leg raising test; VAS, visual analog scale.

**TABLE 2:** Comparison of our 11 cases and 82 cases in literature.

	Our 11 cases	82 cases in literature
Age (year)	49.3 (34–69)	46.8 (17–68)*
Gender		
Female	5 (45.5%)	33/82 (40.2%)
Male	6 (54.5%)	49/82 (59.8%)
Levels		
L1‐2	0	1/82 (1.2%)
L2‐3	0	3/82 (3.7%)
L3‐4	0	7/82 (8.5%)
L4‐5	6 (54.5%)	52/82 (63.4%)
L5‐S1	5 (45.5%)	19/82 (23.2%)
Symptoms and Signs		
Lower back pain	7 (63.6%)	23/36 (63.9%)
Radicular pain	7 (63.6%)	81/82 (98.8%)
Hypoesthesia	7 (63.6%)	22/44 (50.0%)
Muscle weakness	1 (9.1%)	22/44 (50.0%)
SLR		
Ipsilateral	0	1/40 (2.5%)
Contralateral	11 (100%)	27/40 (67.5%)
Both side	0	9/40 (22.5%)
Herniation side		
Right	4 (36.4%)	38/77 (49.4%)
Left	7 (63.6%)	39/77 (50.6%)
Treatment methods		
Surgery	11(100%)	61/82 (74.4%)
No surgery	0	21/82 (25.6%)
Surgical side of LDH		
Ipsilateral side	11 (100%)	37/61 (60.6%)
Contralateral side	0	2/61 (3.3%)
Bilateral sides	0	22/61 (36.1%)
Outcome of surgery		
Significant/complete symptoms relief	11 (100%)	61/61 (100%)
No change/worse	0	0
Outcome of conventional treatment		
Significant/complete symptoms relief	/	15/21 (71.4%)
No change/worse	/	6/21 (28.6%)

*Note*: Continuous variables were reported as mean (range); Categorical variables were expressed as absolute frequency (*n*) and relative frequency (%).

Abbreviations: L, lumbar vertebra; LDH, lumbar disc herniation; SLR, straight leg raising test.

* Five cases were not included to calculate the mean age, because data for each case was not available.

In physical examination, all patients had positive leg‐raising tests for symptomatic sides and negative leg‐raising tests for disc‐herniation sides (Tables 1 and 2). Interestingly, one of them had increased radicular pain of the symptomatic side leg when the straight leg raising test of the disc‐herniation side leg was conducted.

**FIGURE 3:**
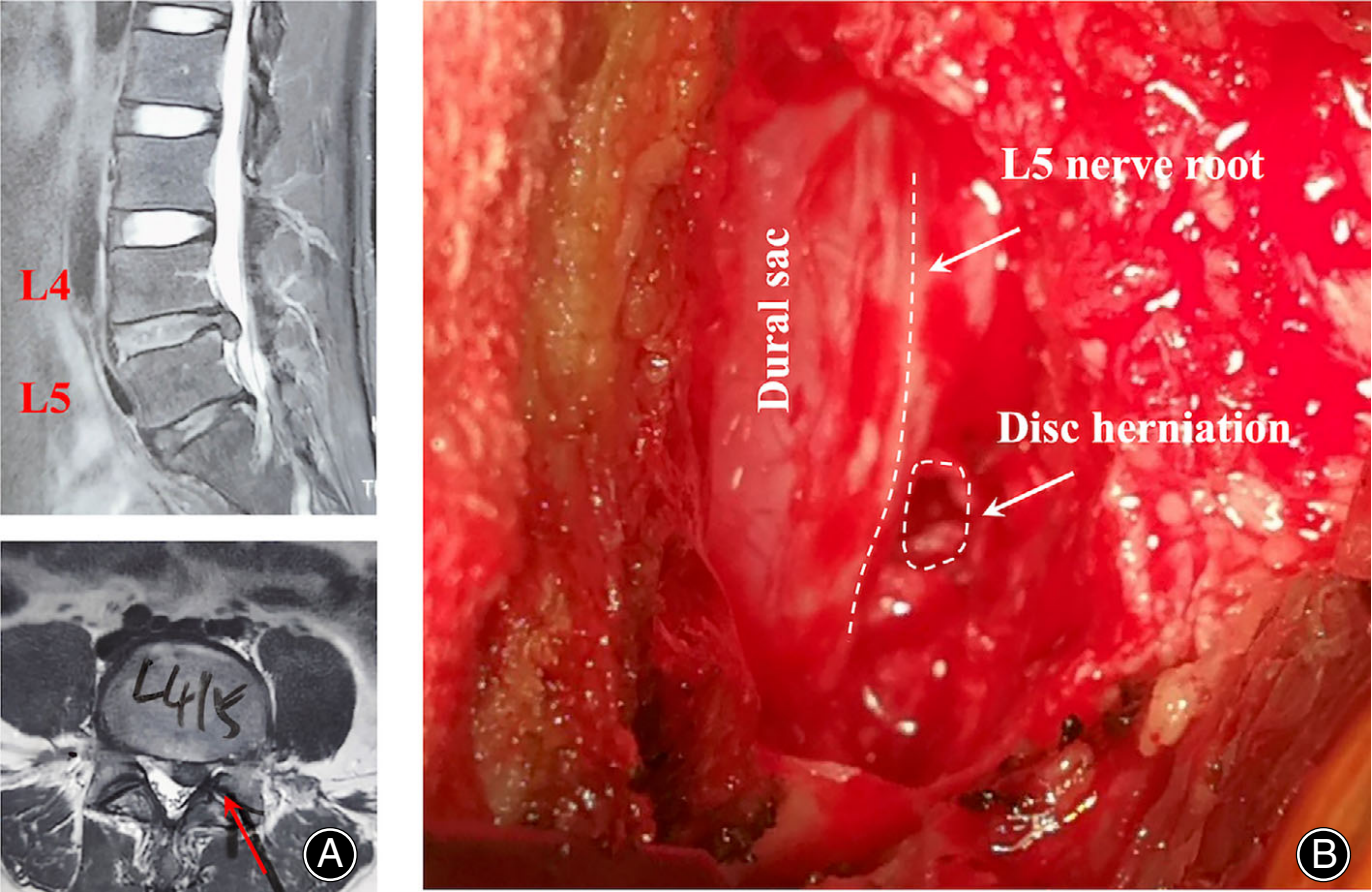
Typical MRI images and intraoperative view of one LDH case with contralateral symptoms. (A) Sagittal and axial MRI images of one LDH case with contralateral symptoms; (B) Surgical findings of one case indicating no direct compression of the ipsilateral neve root by the disc herniation. (LDH, lumbar disc herniation; MRI, Magnetic Resonance Image).

All patients had unilateral LDH, with right‐sided in four patients and left‐sided in seven patients. In our study, no disc hernia was found in L1‐L4. Six cases of disc herniation were located at L4‐L5 and five cases were located at L5‐S1 (Table 2). For these patients, X‐ray plain films showed no spondylolisthesis and lumbar instability; MRI and CT indicated that no disc fragments, osteophytes, epidural fat, or venous congestion were found directly compressing the nerve root on the symptomatic side or the spinal cord. A typical case was presented in Figure 3.

### Treatment and Outcomes

All 11 cases had undergone conservative treatment and failed to relieve symptoms before the surgery. In our series, we chose to perform discectomy and hemilaminectomy only through the herniation side without intervention to the contralateral symptomatic side. During the operation, we observed no change in epidural fat or venous congestion around the nerve on the ipsilateral side. Additionally, in all 11 cases, we found that the lumbar nerve root exited the dural sac either above or below the herniated disc, without directly compressing the ipsilateral nerve root, and therefore, there were no ipsilateral symptoms. After surgery, the radicular pain of all 11 cases significantly improved, as evidenced by a reduction in the VAS (Table 1). At 2–4 years follow‐up, no postoperative complications were observed, and the symptoms of numbness and muscle weakness had significantly improved.

### LDH with Contralateral Symptoms in the Literature

Through searching the published papers in PubMed from 1978 to 2023, 82 cases were reported to have one‐level LDH with contralateral symptoms, 33 were female, and the mean age was 46.8 (range 17–68) (Table 2). Radicular pain (81/82, 98.8%) and lower back pain (23/36, 63.9%) were the most frequently occurring manifestation, followed by hypoesthesia (22/44, 50.0%) and muscle weakness (22/44, 50.0%). According to CT, MRI, or myelography, the LDH was located at L1‐L2 in one case, L2‐L3 in three, L3‐L4 in seven, L4‐L5 in 52, and L5‐S1 in 19. The herniation side of those cases was distributed equally on the left (50.6%) and right side (49.4%). Among 82 cases, only 40 cases provided SLR data, 67.5% of them (27 cases) were positive on the symptomatic side, one was positive on the herniation side, and nine were positive on the bilateral sides (Table 2).

Except for 22 cases that received conservative treatment, most cases (61 in 82, 74.4%) underwent surgeries. Different pathological findings (Table 3) led to different surgery strategies, including the ipsilateral approach (37/61, 60.6%), contralateral approach (2/61, 3.3%), and bilateral approach (22/61, 36.1%). Despite various surgical approaches, all patients who underwent surgery had significant or complete relief of the symptoms. Six of 21 cases reported poor treatment efficacy after continuing conservative treatment (Table 2).

**TABLE 3:** Hypothesis of lumbar disc herniation with contralateral symptoms.

Hypothesis on the presence of contralateral symptoms	Reference
Compression of displaced dural sac	^18^
Compression of sequestrated disc	^10^, ^19^, ^25^
Compression of increased epidural fat	^11^
Compression of venous congestion	^6^
Lateral recess stenosis	^10^, ^15^, ^20^, ^26^
Hypertrophy of facet or ligamentum flavum	^12^, ^17^, ^20^, ^21^, ^22^, ^23^
Inflammation of nerve roots	^14^, ^21^
Traction of dural sac	^7^, ^27^
Disappearance of epidural fat tissue exaggerates friction on nerve root	^14^

Hypothesis on the absence of ipsilateral symptoms	Reference
Anatomical variation of ipsilateral nerve root	
Lower emerging	^18^, ^20^
Absent	^20^

## Discussion

**FIGURE 4:**
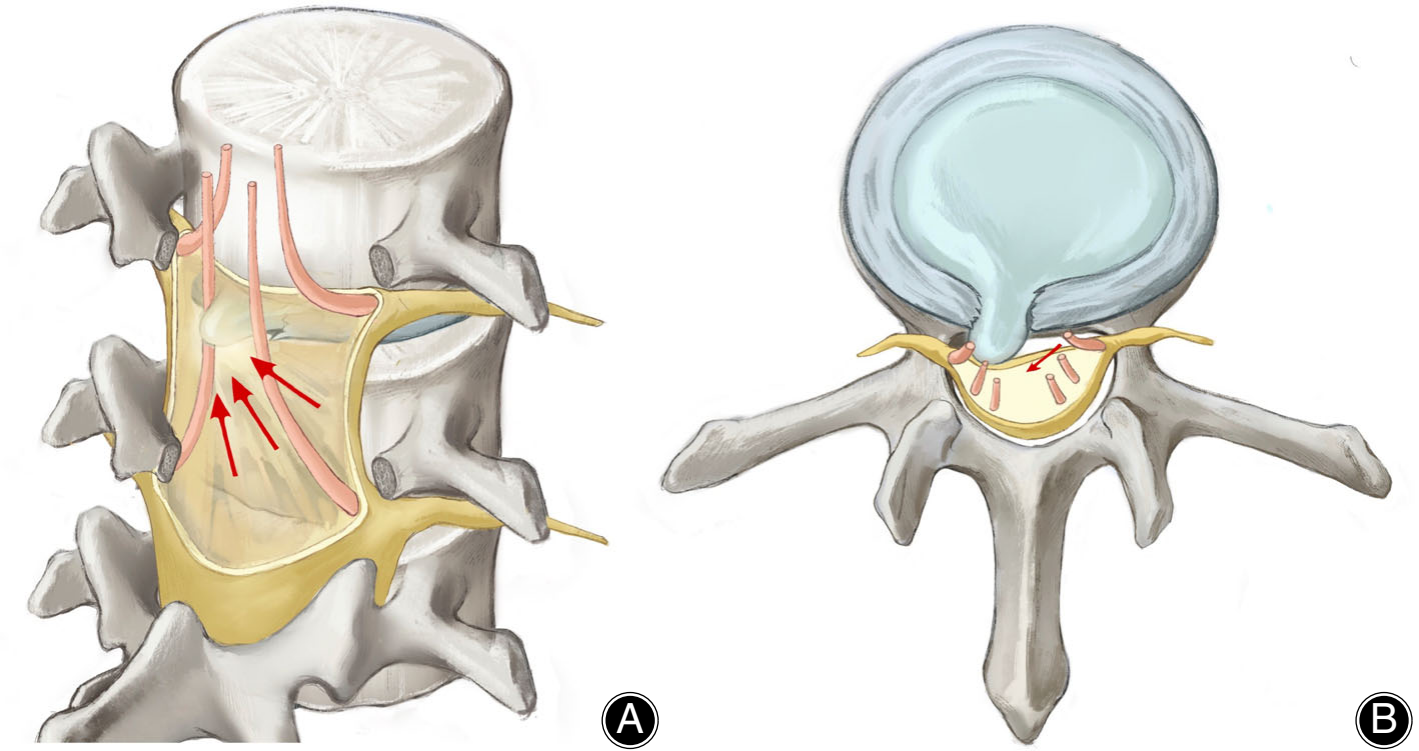
Pathological diagram of LDH cases with contralateral symptoms. (A) A 3D pathological diagram showing that disc herniation does not directly compress the ipsilateral nerve root but tracts the contralateral nerve root via dural mater; (B) A coronary diagram indicating the traction force exerted on the contralateral nerve root by the herniated disc. (LDH, lumbar disc herniation).

This study focused on LDH cases with contralateral symptoms. Upon analyzing our database, we found that the incidence of single‐level LDH with contralateral symptoms without lumbar stenosis, foraminal stenosis on the symptomatic side, scoliosis, and other lumbar operation history was 0.32%. Ipsilateral hemilaminectomy and discectomy surgery on the LDH side achieved good outcomes for all patients. Based on the literature review, surgical approaches involving ipsilateral, contralateral, or bilateral procedures for LDH cases with contralateral symptoms have shown comparable effectiveness. We have also summarized several potential mechanisms for these cases, and hypothesized that traction of the dural sac by the LDH on the contralateral nerve root could explain the contralateral symptoms, while the absence of direct compression on the ipsilateral nerve root could explain the absence of ipsilateral symptoms (Figure 4).

### The Incidence of LDH with Contralateral Symptoms

According to a previous study, the estimated incidence of LDH with contralateral symptoms was 0.02%.^12^ Karabekir et al. reported five LDH cases with contralateral symptoms out of 4170 LDH cases with ipsilateral symptoms in the same period.^12^ However, our single‐level LDH database showed a higher incidence of 0.32% (11/3435) for the mismatching of symptoms and disc herniation, which contrasts with the previous finding. Upon a thorough review of the manuscript, we found that Karabekir used a broad definition of the overall LDH population. All operated patients with signs or symptoms of LDH for more than 15 years were enrolled in their study. In contrast, our study only included adult patients with single‐level LDH, and some of whom were further included for having lumbar stenosis, foraminal stenosis, or scoliosis on the symptomatic side. Therefore, our overall LDH population size was relatively small, which led to an overestimation of the incidence.

### Hypothesis of LDH with Contralateral Symptoms

Nerve root pain in patients with LDH on the ipsilateral side of the lesion is typically attributed to nerve root compression and inflammatory stimulation. However, fewer cases of LDH with contralateral neurogenic pain have been reported, and the underlying pathological mechanism for this mismatch between the herniation and symptoms is still a topic of debate. Several previous hypotheses have been purposed to explain the mismatching of the disc herniation and radicular symptoms (Table 3). Due to the presence of vertebral bodies and spinal ligaments, there is limited space within the spinal canal. A herniated disc takes up the epidural space and pushes out other contents in the canal contralaterally. Albert et al. discovered that the dural sac was displaced on the symptom side and compressed the contralateral nerve root against the pedicle.^18^ The pressure transmitted from the herniation pushes the contralateral nerve root against the vertebral body, the pedicle, the joint, or the ligament. When combined with hypertrophy of facets or ligaments, the nerve is more likely to be stuck in the lateral recess, causing nerve swelling or nerve entrapment.^12^, ^17^, ^21^, ^22^, ^23^

Contralateral lateral recess stenosis has been found in many cases where there is also evidence of nerve inflammation.^10^, ^15^, ^28^ Karabekir et al. compared the thickness of the ligamentum flavum in 200 patients with ipsilateral symptomatic disc herniation with that of five patients with contralateral symptomatic disc herniation alone. They found hypertrophy and asymmetrical thickness (greater thickness on the contralateral side) of the ligamentum flavum in the latter group.^12^

Similarly, increased epidural fat^9^ or venous congestion^6^ will generate compressive forces on the nerve roots. Yang et al. identified the epidural adipose tissue pushed away by the herniated intervertebral disk in their single case reports, assuming that the migrated epidural adipose tissue might put pressure on the contralateral neural contents.^9^ Contrary to Yang, Hayashi et al. revealed the disappearance of epidural fat on the symptomatic side, which they believed weakened the buffering effect on the nerve friction during lower limb movements, and caused “friction radiculitis” on contralateral nerve roots.^14^ Kalemci et al. reported a left‐sided LDH patient with ipsilateral radicular pain and contralateral foot drop. Surgeons found that the right L5 nerve root was compressed by prominent venous engorgement and congestion.^6^ Bipolar cauterization of the venous plexus improved the neurological deficits on the right side. No specific reason was found for the venous congestion yet. After rising from the spinal cord, the lumbar nerve roots pass through the intervertebral foramen, accompanied by intervertebral veins. Congested intervertebral veins result in foraminal stenosis and spinal nerve compression. In addition to vascular lesions, venous congestion could be pathological changes secondary to any imbalance of volume or distribution of spinal contents.

Sequestrated herniation can develop radicular symptoms on the side opposite to the herniation.^10^, ^19^, ^26^ Many researchers found fragments of discs on the symptomatic side of the patient. For example, Branam et al. reported that a huge fragment of the right L5‐S1 herniated disc migrated to the left anterolateral epidural space and touched the left L5 nerve root.^26^

In some patients, there is no evidence of nerve compression on the symptomatic side and the traction force plays a bigger role.^7^, ^17^, ^24^, ^28^ According to Sucu and Gelal, the top of the herniated disc (five cases) protruded towards the ipsilateral side on imaging studies, thus there was unequal traction force generated on both sides of the nerve root.^7^ The contralateral nerve was subjected to a greater force theoretically.^7^ Akdeniz et al. supplemented that more dural attachment on the posterior longitudinal ligament would fix the nerve root and put it under greater strain.^28^

As mentioned above, the proposed hypotheses were based on their case‐specific pathological findings, which may not be related to the LDH. Also, the sample size was relatively small. To address the limitations, we selectively collected 11 LDH cases with contralateral radiculopathy while excluding cases with additional lumbar degenerative changes such as lumbar stenosis, foraminal stenosis on the symptomatic side, multilevel disc herniations, and scoliosis. By doing so, we aimed to remove other potential confounding factors and better understand the relationship between LDH and contralateral radiculopathy.

In our 11 cases, no epidural fat or venous congestion was responsible for the contralateral symptoms. Instead, we propose that traction on the contralateral nerve root due to the herniated disc may explain the observed radicular pain. Based on the surgical findings of our 11 LDH cases with contralateral symptoms, we hypothesized that it is silence on the lesion side because the corresponding nerve root exited the dural sac below the herniated intervertebral disc. However, the herniated lumbar disc pushed the dural mater and then pulled the dural sheath of the contralateral nerve root, leading to the exhibited contralateral symptoms (Figure 4).

Few pieces of literature discussed the absence of ipsilateral symptoms. Consistent with our results, patients often have anatomical variants of the spinal nerve root on the herniated side, such as a lower emerging position,^18^ transverse course, and nerve root missing.^20^ These factors prevent the disc from reaching the nerve root and producing nerve root irritation. Whether it would be the same for every patient needs further verification.

To be noted, one of our cases showed a positive “crossed straight leg raising test” (or Fajersztajn's test), which was first described by Fajersztan in 1977.^25^ According to Karl, pain on the lesion side during the rise of the healthy side leg indicates a disc rupture.^29^ Large and central LDH can also result in a positive Fajersztajn's test on the healthy side.^30^ Nevertheless, in our case, increased pain was observed on the unaffected side during the “crossed straight leg raising test” presented on the herniated side. Sciatic nerve roots can move 2–8 mm at the intervertebral foramen in a straight leg raising test.^31^ A possible explanation for this might be that the nerve root and the dural sac on the symptomatic side are pulled further to the opposite side when the herniated‐side leg is raised giving rise to increased pain on the symptomatic side.

### Comparison of Surgical Treatment for LDH with Contralateral Symptoms

The decision to perform surgery in the case of radicular leg pain accompanied by contralateral disc herniation without significant abnormality on the ipsilateral side is currently a dilemma for surgeons. Surgeons may be struggling to ascertain whether or not the presenting symptoms are related to the herniated disk. In this situation, the surgeon is reluctant to operate due to the risk of failure of surgery and complications. Therefore, long‐term conservative treatment and delayed surgeries often occur in these patients. We certainly do not advocate for the removal of every contralateral lesion, but if conservative treatment fails or the symptoms heavily affect the patient's daily life and no other reasons can explain the patient's radicular symptoms except contralateral LDH, surgery may be considered.

Moreover, when spinal surgeons decide to conduct an operation for LDH cases with contralateral radicular symptoms, they may hesitate to choose the surgical site—compressive side, symptomatic side, or both. Although surgical interventions are recommended to be performed on the symptomatic side according to the conventional approach, some surgeons recommend performing surgery on the disc‐herniation side or a bilateral operation under particular circumstances on the symptomatic side such as the lateral recess stenosis, hypertrophy of facets and ligaments, or free‐floating debris of the herniated disc. Our finding indicates that discectomy via the herniation side could successfully eliminate the patient's contralateral radicular symptoms. This is consistent with previous publications. Among 53 LDH cases with contralateral symptoms, 56.6% received discectomy via only herniation side and achieved success. However, few surgeons, as evidenced by rates of surgery, support discectomy via unilateral ipsilateral to the symptomatic side.^9^ Besides, in these cases, significant nerve root compression on the symptomatic side was found during the operation. For most LDH patients with contralateral symptoms, the effect of prophylactic removal of tissue in epidural space remains to be elucidated. In addition to that, discectomy via bilateral laminectomy requires a longer surgery time and has higher risks of complications. Evidence from surgical interventions supports treating LDH with contralateral symptoms through discectomy via unilateral ipsilateral to herniation side. Indeed, our 11 cases also validate this. Bilateral laminectomy and discectomy should be performed when strong evidence indicates the compression of the contralateral nerve root. With the development of minimally invasive surgery, endoscopic surgery has demonstrated similar effectiveness in treating LDH with contralateral symptoms^10^, ^11^, ^14^, ^23^ when compared to conventional methods. As endoscopic surgery causes less trauma, we believe that it will become a widely adopted treatment option for such cases in the future.

### Strengths and Limitations of the Study

In this study, we reported 11 LDH cases with contralateral symptoms and 55 cases from the literature to summarize the clinical features, treatment, outcomes, and hypotheses. We first reported the incidence of LDH with contralateral symptoms among simple and single‐level LDH patients without lumbar stenosis, foraminal stenosis, scoliosis, and a history of lumbar operation. The study also had some limitations. As a retrospective study, there might be selection bias in the process of data collection. Moreover, we designed strict screening criteria, which led to a relatively small sample size and poor presentation of the corresponding population. Although our study had a relatively large sample size compared to similar studies, there is a need for future multicenter studies with stronger evidence to address these limitations and provide more robust conclusions.

### Conclusion

In conclusion, the mismatching of symptoms and disc herniation of LDH can be explained by the anatomical features of lumbar nerve roots. Unilateral‐approach surgery ipsilateral to herniation can effectively treat LDH patients with contralateral symptoms. Bilateral laminectomy is needed if the compression of the contralateral nerve root exists. Future studies need to be conducted to further elucidate the impact of unilateral‐approach surgery ipsilateral to symptomatic side.

## Author Contributions

All authors made substantive contributions in this study, and can be qualified as authors according to The International Committee of Medical Journal Editors (ICMJE). Conceptualization—Qingyang Gao and Huiliang Yang; Methodology—Qingyang Gao; Investigation—Huiliang Yang; Formal Analysis—Qingyang Gao and Huiliang Yang; Resources—Yueming Song; Writing—Original Draft, Qingyang Gao and Huiliang Yang; Writing—Review & Editing, Umar Masood and Huiliang Yang; Visualization—Qingyang Gao and Ying Cen; Supervision—Chunguang Zhou and Ying Cen; Funding Acquisition—Huiliang Yang and Chunguang Zhou. All authors are in agreement with the manuscript.

## Funding Information

This work was supported by grants from Sichuan Science and Technology Program (2022YFS0260), National Natural Science Funds of China (82102521), Science and Technology Project of the Health Planning Committee of Sichuan (21PJ036).

## Conflict of Interest Statement

The author declared no conflicts of interest.

## Ethics Statement

This study was approved by the Institutional Ethics Committee of West China Hospital, Sichuan University (2019–852). All patients provided written informed consent for publication in this study.

## Authorship Declaration

This declaration acknowledges (1) that all authors listed meet the authorship criteria according to the latest guidelines of the International Committee of Medical Journal Editors and (2) that all authors read and approved the final manuscript.

## Supporting information


**Table S1.** Variables extracted from related LDH cases.

## Data Availability

The datasets used and/or analyzed in this study are available from the corresponding author on reasonable request.
